# Novel Statistical Approaches for Non-Normal Censored Immunological Data: Analysis of Cytokine and Gene Expression Data

**DOI:** 10.1371/journal.pone.0046423

**Published:** 2012-10-26

**Authors:** Nikolaus Ballenberger, Anna Lluis, Erika von Mutius, Sabina Illi, Bianca Schaub

**Affiliations:** University Children's Hospital Munich, Department of Pulmonary and Allergy, LMU Munich, Munich, Germany; University of Westminster, United Kingdom

## Abstract

**Background:**

For several immune-mediated diseases, immunological analysis will become more complex in the future with datasets in which cytokine and gene expression data play a major role. These data have certain characteristics that require sophisticated statistical analysis such as strategies for non-normal distribution and censoring. Additionally, complex and multiple immunological relationships need to be adjusted for potential confounding and interaction effects.

**Objective:**

We aimed to introduce and apply different methods for statistical analysis of non-normal censored cytokine and gene expression data. Furthermore, we assessed the performance and accuracy of a novel regression approach in order to allow adjusting for covariates and potential confounding.

**Methods:**

For non-normally distributed censored data traditional means such as the Kaplan-Meier method or the generalized Wilcoxon test are described. In order to adjust for covariates the novel approach named Tobit regression on ranks was introduced. Its performance and accuracy for analysis of non-normal censored cytokine/gene expression data was evaluated by a simulation study and a statistical experiment applying permutation and bootstrapping.

**Results:**

If adjustment for covariates is not necessary traditional statistical methods are adequate for non-normal censored data. Comparable with these and appropriate if additional adjustment is required, Tobit regression on ranks is a valid method. Its power, type-I error rate and accuracy were comparable to the classical Tobit regression.

**Conclusion:**

Non-normally distributed censored immunological data require appropriate statistical methods. Tobit regression on ranks meets these requirements and can be used for adjustment for covariates and potential confounding in large and complex immunological datasets.

## Introduction

The understanding of immunological mechanisms underlying human disease has largely increased over the last decades. In this context, different regulatory mechanisms involving gene expression at messenger ribonucleic acid (mRNA) and protein level (of e.g. cytokines) play an important role. When a complex cascade of regulatory immune mechanisms becomes initiated in a healthy or diseased individual, this leads to gene transcription, which can be measured as gene expression on mRNA level. Following activation of subsequent regulatory mechanisms including signaling pathways, cytokines can get released by different cell types, which can be measured as protein expression. Cytokines reflect mediators of different immune and inflammatory reactions. In order to take complex relationships of immune regulation, interplay of several pathways and external influencing factors such as environment into account, analysis that controls for confounding and interaction is crucial.

Our birth cohort study Paulchen aims to assess the influence of environmental lifestyle factors and genetic background on neonatal immune maturation in the development of atopic diseases [1]. Among other immune parameters we have assessed cytokine secretion both at mRNA level by real-time RT-PCR (gene expression) and at protein level by LUMINEX technology. However, collected data from both measurements have certain characteristics that are typical for cytokine expression data, which generally make statistical analysis challenging [2]. Data are often not normally distributed and transformation into a distribution on which parametric tests can be applied e.g. log-transformation, is often not successful. Another common characteristic is that cytokine datasets may contain non-detectable values and thus the issue of “censored data” needs to be addressed. When protein expression is assessed by LUMINEX technology early in life, when many markers of immune regulation are expressed at low concentrations, left censoring at a single censoring level can occur. If cytokine concentrations are below detection threshold, they are not quantifiable any more. On the other hand, “right censored” data may occur when concentrations exceed a certain threshold on the top of the measurement scale and thus levels are also not exactly quantifiable. Right censoring at multiple censoring levels may occur when genes or cytokines are measured by real-time RT-PCR at mRNA level and are expressed in relation to a given housekeeping gene by the Δct formula [3]. However, in contrast to left censoring due to measurement of low protein concentrations by LUMINEX technology, right censoring in the context of measurement by real-time RT-PCR is less obvious.

The literature recommends different methods dealing with censored or “non-detectable” data. These suggestions comprise substitution of the values above or below the detection level [4], Tobit regression [5], multiple imputation [6], [7] and deletion [8] among others. Yet, simple substitution is not advisable as it may lead to strongly biased results [9]. Other methods like Tobit regression require strong parametric assumptions which can rarely be fulfilled by cytokine data [10], [11]. Multiple imputation has been shown to be valid [6], [7] but is quite time consuming, especially when data sets are large. Furthermore, multiple imputation is not supported by all statistical packages.

Additionally, complex relationships present between and within immunological and epidemiological parameters have to be taken into account by appropriate statistical models in order to avoid confounding and to reveal interaction effects.

Therefore it is important to use statistical tools that i) take censoring into account, ii) are not prone to violating parametric assumptions, iii) allow adjusting for covariates, potential confounding and interaction effects and iv) are available in common statistical packages.

The first aim of this paper was to demonstrate by means of an example using immunological data how to statistically analyze non-normal censored cytokine data and furthermore illustrate the consequences when these data are analyzed by inappropriate statistical methods that do not take censoring into account. Here we apply traditional established methods, which are mainly applied for survival studies. Our example addresses a typical immunological research question where gene expression is dependent on a certain diseases status (e.g. asthma).

The second aim was to evaluate a novel regression approach, named Tobit regression on ranks, for non-normally distributed censored data in order to allow adjusting for potential confounding and interaction effects. This is relevant for studies on relationship between disease status and gene expression, when adjustment for other variables (epidemiological parameters, e.g. gender or age) is required. The performance and accuracy of the Tobit regression on ranks was evaluated by a simulation study and a statistical experiment using permutation and bootstrapping.

## Methods

### 1. Data

The data used in this study are from the Paulchen Study, a birth cohort study performed in southern Germany [1]. In addition, data were simulated, permutated and bootstrapped in order to address performance and accuracy.

### 2. Statistical analysis for censored cytokine data from LUMINEX and RT-PCR technology

- Summary statistics are computed by standard methods for left censored data and by the Kaplan-Meier method for right censored data. The Kaplan-Meier method was applied to an example with right censored RT-PCR cytokine data. Statistical testing of differences between two or more groups (without adjusting for covariates) is done by the Kruskal-Wallis-Test for left censored data and the generalized Wilcoxon test for right censored data. The generalized Wilcoxon test was applied to an example with right censored RT-PCR cytokine data.
- For non-normally distributed censored data which need to be adjusted for potential confounding and interaction effects, we introduce the new approach Tobit regression on ranks. The method was described and its performance was evaluated by a simulation study. We defined performance as Type-I error rate and power. Therefore, we simulated 10000 data sets by drawing samples from a hypothetical population similar to the PAULCHEN study with respect to sample size and proportion of censored observations. We simulated a two group comparison with an interval-scaled dependent variable. We assumed normal error distribution as we aimed to include the Tobit regression as gold standard in our simulation study (details in Supplement S1).

To address accuracy, analysis including a simple two group comparison was performed. In analogy to left censored heavily skewed cytokine data, we created a variable by transformation of a normally distributed variable into a heavily skewed one with similar properties as typical skewed cytokine data (Fig. S1 and Fig. S2). As measure of accuracy the balanced accuracy was used: 
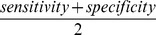
 = 
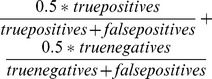
 [12]. Specificity was assessed by permutating (resampling without replacement) the grouping variable 1000 times. By applying this method the known systematic group effect is removed. Sensitivity was achieved by bootstrapping. Bootstraps allow the experiment to be repeated statistically by randomly drawing samples out of the original data with replacement. The higher the value of balanced accuracy the more valid is the applied statistical model. A value of 0.5 is the lowest possible value (Supplement S1).

## Results

### 1. Cytokine and gene expression data are subject to censoring

**In the following the two types of skewed immunologic data are described which were analysed in this study:**

Concentrations below a certain detection threshold (non-detectable) are called left-censored with one “detection limit”. Measurements of secreted proteins (e.g. cytokines from serum or supernatants) by LUMINEX technology or ELISA represent a typical example. For evaluation the concentration has to exceed a certain detection threshold determined by the respective laboratory technique, e.g. a given cytokine such as interferon-γ may have a detection level of 1.3 pg/ml given by the manufacturer's instructions. Consequently, all measurements below 1.3 pg/ml are left censored at one detection level. In this situation censoring is obvious.

**Figure 1:**
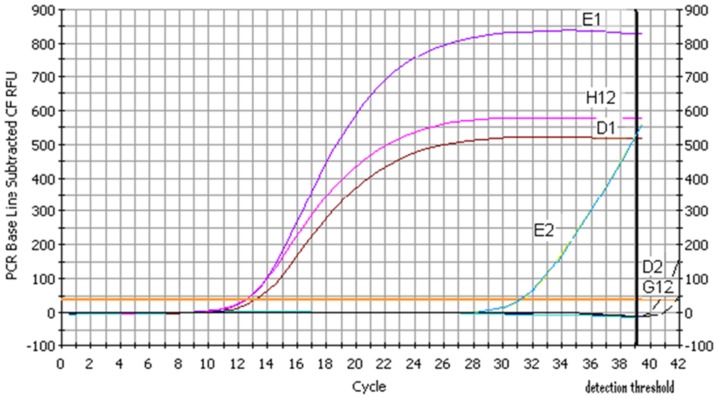
Right censored mRNA expression data with multiple censoring levels. E2, G12, D2 (genes of interest) are set relative to housekeeping genes D1, E1 and H12. D2 and G12 are not quantifiable (right censored).

In contrast to left censoring at one detection level right censoring at multiple detection levels can occur in cytokine and other gene expression measurements when they are assessed at mRNA level by real-time RT-PCR. In this situation censoring is less obvious. During real-time RT-PCR, amplification of gene expression is detected by an increase of the fluorescence signal. The cycle threshold (ct) is the number of PCR cycles required for the fluorescence signal to exceed the detection threshold set to the log-linear range of the amplification curve. In Fig. 1 the ct value for the gene of interest E2 is 31.1. If the ct exceeds the maximum number of cycles specified by the real-time PCR program the value is considered to be very low and not-quantifiable (comparable to non-detectables using LUMINEX technology), e.g. ct values of G12 and D2 in Fig. 1. For inclusion of not-quantifiable values in the analysis, the maximum amount of cycles predetermined in the PCR program is assigned as ct (e.g. 39 cycles). The genuine ct value of a not-quantifiable PCR product is actually greater than the assigned ct (in Fig. 1 approximately 39). Thus, ct values in G12 and D2 are right-censored. The determined ct from the gene of interest is calculated relative to the ct value of a housekeeping gene according to the formula [13] 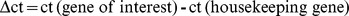. Housekeeping genes used for normalization are expressed at relatively constant levels. In Fig. 1 housekeeping genes are represented as D1, E1 and H12. If the ct from the gene of interest is right censored, the resulting Δct contains a right censored value and has multiple quantification (censoring) levels. A numerical example taken from Fig. 1 is illustrated in Table 1 where we consider the determined cycle threshold as 39. A higher Δct indicates lower mRNA expression.

**Table 1:** Example of right censored mRNA expression data with multiple censoring levels.

Case	Gene of interest	CT of gene of interest	Gene detection	Housekeeping gene (HKG)	CT of housekeeping gene	Δct value	Censored Δct value	Interpretation of Δct value
A	E2	31.1	yes	H12	12.6	18.5	no	18.5
B	G12	>39	no	H2	12.4	26.6	yes	>26.6
C	D2	>39	no	D1	13.1	25.9	yes	>25.9

Case A: The threshold cycle of the gene of interest E2 for case A is 31.1. The threshold cycle of the housekeeping gene (HKG) H12 is 12.6. The Δct value is 18.5 (31.1–12.6 = 18.5). In this case no censoring is present.

Case B: The threshold cycle of the gene of interest G12 for case B exceeds 39, is not quantifiable any more, and consequently, is right censored. The threshold cycle of HKG H2 is 12.4. The Δct value is 26.6 (39–12.4 = 26.6). The Δct value contains the right censored value of 39, thus the resulting Δct value is also right censored. Consequently, the Δct value has to be interpreted as greater than 26.6.

Case C: The threshold cycle of the gene of interest D2 for case C exceeds 39, is not quantifiable and also right censored. The threshold cycle of HKG D1 is 13.1. The Δct value is 25.9 (39–13.1 = 25.9). The Δct value contains the right censored value of 39, thus the resulting Δct value is also right censored. The Δct value has to be interpreted as greater than 25.9. However, as the threshold cycle of the HKG is different to case B (26.9 vs. 25.9) the resulting right censored Δct value has a different censoring level than in case B.

Thus, Δct values may be right censored data at different censoring levels.

### 2. Summary statistics for censored data: description of approach and its application

When computing summary statistics such as the median for left censored protein expression measured e.g. by LUMINEX technology, standard methods as implemented in all statistical packages (e.g. *Proc univariate* in SAS [14]) are applicable. As there is generally only one detection level per variable there is no loss of information if all values below the detection level are assigned tied ranks [9]. Based on ranks the median can be computed and its corresponding confidence intervals (CI) can be derived from a table with a continuous distribution based on order statistics [9]. In contrast to the interquartile range (IQR), which represents the dispersion in the study sample, the confidence interval gives an estimated range of values which is likely to include the parameter of interest (e.g. mean or median) in the population.

**Figure 2:**
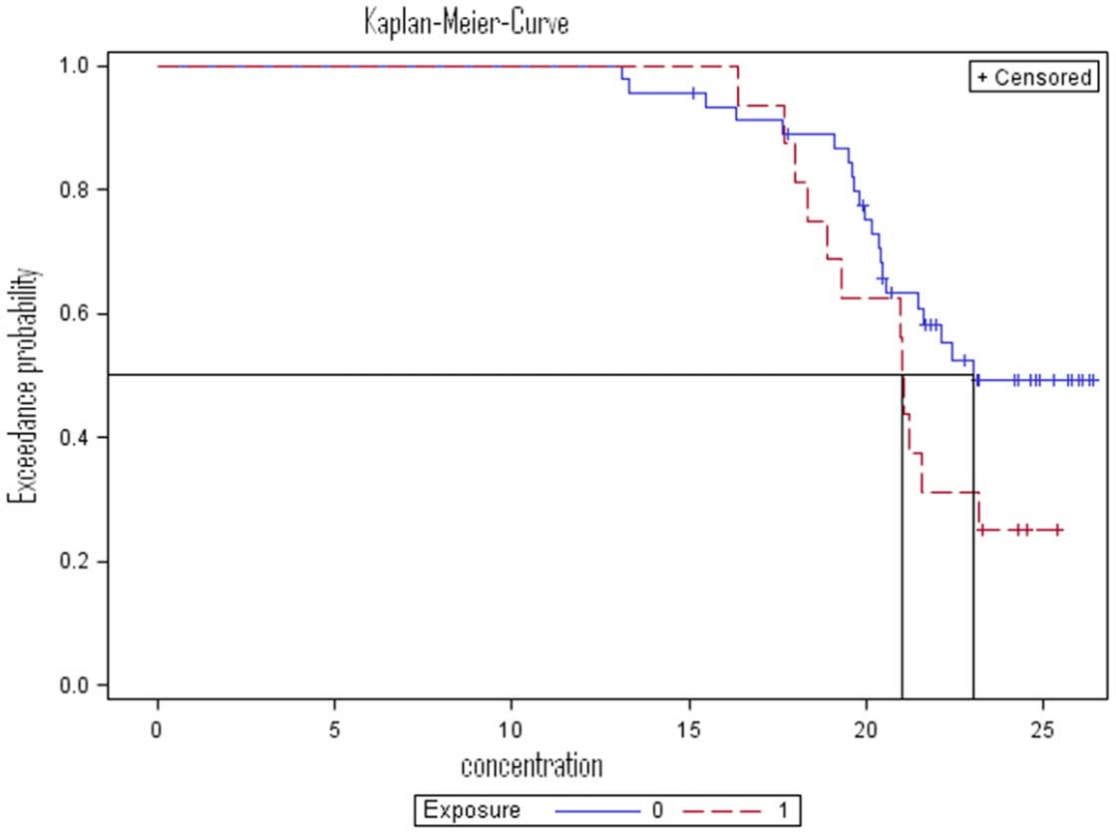
Survival function plot (Kaplan-Meier curve) of a cytokine depending on a certain exposure expressed as Δct and assessed by real time RT-PCR. Right censoring with multiple detection levels is present.

In contrast to left censoring at one single detection level, for analyzing right censored gene expression data the Kaplan-Meier method should be used [15]. This is the standard method for producing summary statistics of right censored survival data at multiple detection levels by calculating the survival probability. For immunological analysis, instead of survival probability rather the probability of exceeding a certain gene expression is relevant. However, the underlying idea is similar. In Table S1 we illustrate in detail how a life table according to the Kaplan-Meier method is created to compute summary statistics for Δct values using an example of immunological data. We aimed to assess whether gene expression (Δct) of a certain cytokine is dependent on a certain exposure (Table 2). Data were non-normally distributed and could not be transformed to a valid distribution. From the life table based on the Kaplan-Meier method estimates like the median, IQR and percentiles were computed and visualized by plotting the Kaplan-Meier function (Fig. 2). The plot presents the probabilities of exceeding a certain gene expression level depending on the exposure. The median is obtained by drawing a horizontal line at 0.5 on the vertical axis and drawing a perpendicular line to the x-axis at the points where the horizontal line intersects the Kaplan-Meier function. The corresponding confidence intervals are computed according to Greenwood's formula [9].

**Table 2:** Characteristics of an immunological example with gene expression (IL-22-LpA) expressed as Δct and assessed by real time RT-PCR.

Exposed	Sample Size	Number right Censored	Percent right Censored
no	40	11	27.50
yes	13	1	7.69
total	53	12	22.64

To illustrate the consequences when censoring at multiple detection levels of Δct data is not taken into account we compared the results derived from the Kaplan-Meier curve with those from the standard method that can only be used for censoring at one single detection level. Summary statistics (Table 3, median with confidence interval) of the exposed group produced by either method are identical (either method: median 18.40 (confidence interval: 15.30; 20.85). However, in the unexposed group, the median and the upper bound of the corresponding confidence interval produced by the Kaplan-Meier method were greater than those produced by the standard method (Kaplan-Meier: 20.15 (18.70; 21.85); standard method: 19.70 (18.70; 20.85)). The different results are explained by the fact that the Kaplan-Meier method captures the information of the proportion of censored observations in the data which is ignored by the standard method.

**Table 3:** Summary statistics and p-values for gene expression (Δct) by methods that ignore and take censoring into account.

N° per group	Standard method	Kaplan-Meier method	Wilcoxon	Generalized Wilcoxon	Tobit on ranks
	Median (CI)		p-value		
Non-exposed: 40	19.70 (18.70–20.15)	20.15 (18.70–21.85)	0.18	0.06	0.06
Exposed: 13	18.40 (15.30–20.85)	18.40 (15.30–20.85)			

### 3. Statistical testing of differences between two or more groups: description of approach and its application

When analyzing non-normally distributed and left censored cytokine expression data (e.g. LUMINEX technology), standard non-parametric rank-based tests as the Wilcoxon rank-sum-test [16] or the Kruskal-Wallis-Test [17] may be applied without loss of information [9]. As there is generally only one detection level per cytokine there is no loss of information if to all values below (or above) the detection level are assigned tied ranks.

When analyzing differences between two or more groups of gene expression data that are right censored at multiple detection levels (RT-PCR data expressed as Δct), statistical tests which capture information in the censored proportion of the Δct data are required. The generalized Wilcoxon test [18], [19], a modification of the Wilcoxon rank-sum test, estimates the U-score of each censored value according to the survival function of the previously uncensored observation. Basically, the test investigates whether survival functions in two or more groups differ significantly. In SAS the *Proc lifetest* procedure covers the generalized Wilcoxon test. To illustrate the consequences when censoring of Δct data is not considered, results from the generalized Wilcoxon test and from standard Wilcoxon test were compared when applied to the above mentioned example (Δct) with right censoring at multiple detection levels (Table 3). The p-value given by the generalized Wilcoxon test was borderline significant whereas the standard Wilcoxon test missed the effect (p-value: 0.06 vs. 0.18). Again, the results differ due to the fact that the generalized Wilcoxon test captures the information of the proportion of censored observations in the data which is ignored by the standard method.

### 4. Tobit regression on ranks: a model for non-normal distributed censored data

The generalized Wilcoxon test does not allow adjustment for covariates and potential confounding. Hence, a regression technique as the Tobit regression is required to assess the simultaneous effect of different variables on the outcome. However, the Tobit regression is based on strong statistical assumptions. Violating these assumptions may lead to biased results [10], [11]. Based on the concept of Iman and Conover [20]–[22] we ranked our data from lowest to highest and performed the Tobit regression on ranks (Mathematical details in Supplement S1). Rank transformation procedures lead to distribution free tests. We conducted a simulation study to assess the type-I and type-II error rate in order to evaluate the performance of the Tobit regression on ranks comparing to other statistical methods namely quantile regression, logistic regression, the Wilcoxon rank sum test and the classical Tobit regression. We considered the classical Tobit regression as the gold standard for censored regression analysis. Thus, we assumed normal error distribution. However, in reality normality or log-normality as an underlying distribution is rarely found.

#### a) Results of the simulation study: type-I error rate and power of the Tobit regression on ranks is comparable to the classical Tobit regression

**Figure 3:**
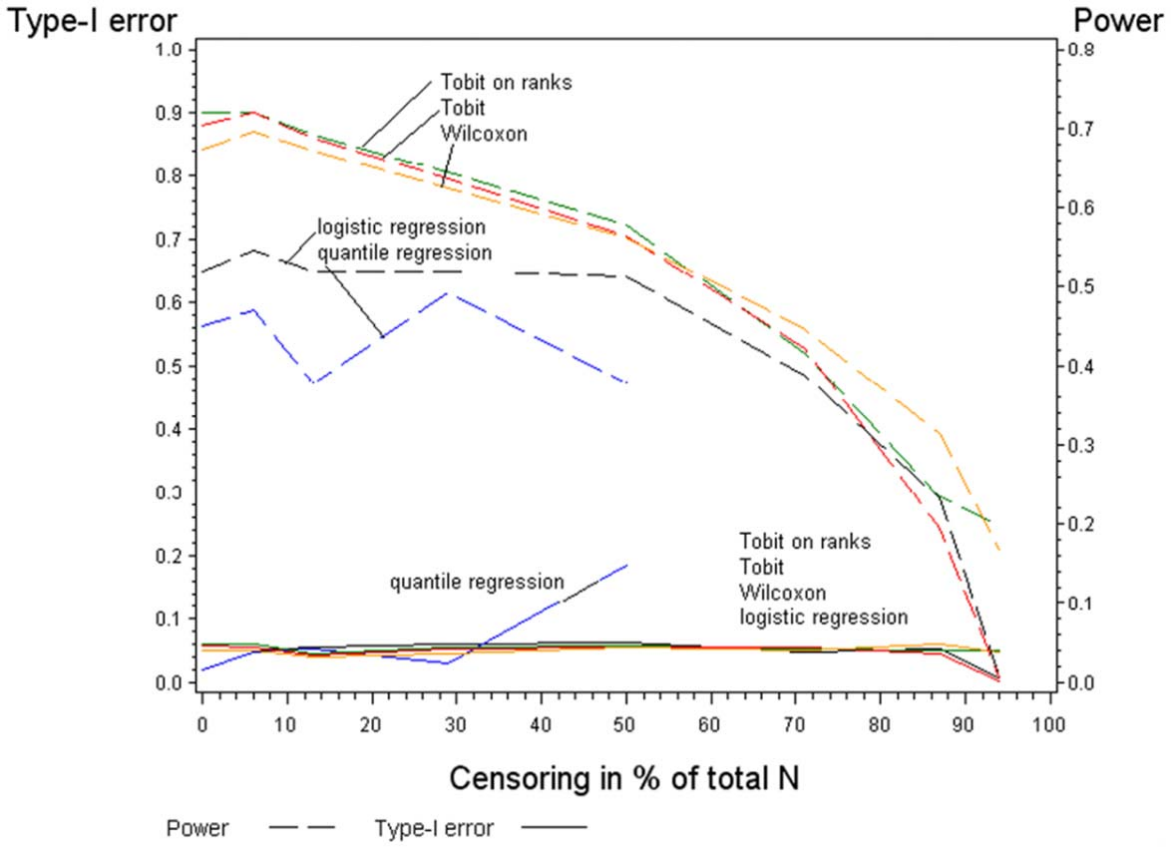
Power and type-I error rate for five statistical methods with N = 50 in each group.

The results of the simulation study are presented in Fig. 3 with N = 50 in both groups. With respect to type-I error all models and tests apart from quantile regression performed equally well with increasing proportion of censoring. When the amount of censoring reached 50% the quantile regression produced higher rate of false positive results (approximately 20%). With more than 50% of censoring quantile regression, when based on the 50^th^ quantile (median), was no longer applicable.

With respect to power the Wilcoxon test, the Tobit regression and the Tobit regression on ranks performed equally well until approximately 70% of censoring. The power slightly decreased with increasing amount of censoring from app. 87% (0% censoring) to approximately 55% (70% censoring). With higher amount of censoring the Wilcoxon test produced the smallest false negative rate. However, the Wilcoxon test can neither control for confounding nor for interaction effects. Both logistic regression and quantile regression had little power especially with less than 50% of censoring.

#### b) High accuracy of the Tobit regression on ranks when applied to heavily skewed data

**Figure 4:**
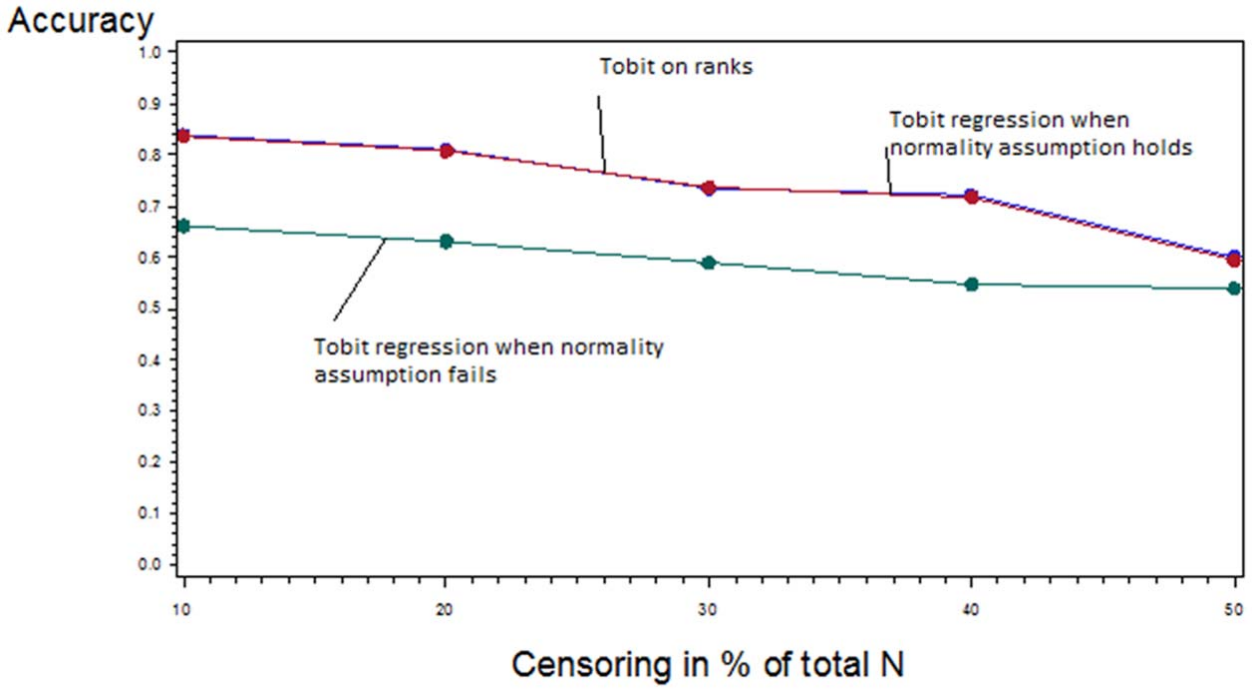
Accuracy of Tobit on ranks performed on heavily skewed variable, Tobit on normally distributed variable and Tobit on heavily skewed variable depending on proportion of left censoring. Tobit on ranks performs equally accurate as Tobit regression on normally distributed variable. When Tobit is applied to heavily skewed variable its accuracy is strongly reduced.

The accuracy of the Tobit regression on ranks applied to a highly skewed and left censored variable (see Fig. S2) is compared to the accuracy of the classical Tobit regression when applied to the same normally distributed and left censored variable (Fig. 4). Depending on increasing proportions of censoring, accuracy decreases with increasing proportion of censoring for all methods. However, the accuracy of the Tobit regression on ranks is almost identical with the accuracy of the classical Tobit regression when the assumption of normality holds (red and blue line, Fig. 4)

Furthermore, the accuracy is depicted when the classical Tobit regression is applied to the highly skewed and left censored variable (green line, Fig. 4). In this case, the parametric assumption of normality fails. As expected, the accuracy is much lower.

In summary, the performance and the accuracy of the classical Tobit regression and the Tobit regression on ranks were comparable. However, the great advantage of the Tobit regression on ranks is its applicability to non-normally distributed data.

### Application of the Tobit regression on ranks on Δct data

The Tobit regression on ranks was applied to the above mentioned data (Δct) (Table 3). While the Wilcoxon test and generalized Wilcoxon test differ strongly (p = 0.18 vs. p = 0.06) the Tobit regression on ranks produced p-values of similar magnitude compared with the generalized Wilcoxon test (p = 0.06) confirming the appropriateness of Tobit regression on ranks.

## Discussion

Analysis of complex immunological datasets requires novel statistical approaches. In this study, we have shown that for analysis of non-normally distributed and censored data computation of summary statistics, comparison of group differences and adjusting for covariates have to be conducted by appropriate methods to avoid biased estimates.

We have shown that in the case of left-censoring, standard techniques for computing summary statistics and testing group differences may be applied. In contrast, our results show that in order to compute summary statistics (e.g. median and confidence interval) and test group differences we applied the Kaplan-Meier method and the generalized Wilcoxon test respectively using gene expression data (shown as Δct) with right censoring. Both summary statistics and p-values from the exposed group stayed unaffected independent of taking censoring into account. This was due to a small proportion of censored observations in this group. In contrast, with greater proportion of censored observations in the unexposed group both summary statistics (median and confidence interval) and the p-values differed more strongly between methods (Kaplan-Meier method and generalized Wilcoxon test vs. standard methods) that do or do not take censoring into account. Both the Kaplan-Meier method and the generalized Wilcoxon test captured the information of the censored proportion in the data. Consequently, in the setting of censored cytokine and gene expression data applying specific methods is crucial.

Immunological data sets often contain huge number of variables aiming to reveal complex relationships among immunological parameters in combination with influencing factors (e.g. environment). Therefore, appropriate statistical models are of high importance [2]. Proposed recommendations are often not applicable: substitution of the values above or below the detection level [4] may lead to strongly biased results. Tobit regression is prone to violating parametric assumptions [10], [11] and multiple Imputations [6], [7], which is a valid alternative, may be time consuming and is not supported by all statistical packages. The Tobit regression on ranks does not require parametric assumptions, takes censoring into account, allows adjusting for covariates and potential confounders and is available in common statistical packages. In our simulation study the performance of the Tobit regression on ranks with respect to power and type I error rate was comparable to both classical Tobit regression and Wilcoxon test over different sample sizes and varying amounts of censoring. In comparison to logistic regression and quantile regression, the Tobit regression on ranks performed much better. The fact that logistic regression has less power than other models is due to dichotomisation, resulting in loss of information [6]. We have also shown that quantile regression had even less power and could be only applied up to 50% of censoring. Thus, both logistic regression and quantile regression cannot be recommended. Additionally, we confirmed the good performance of the Tobit regression on ranks shown in the simulation study by applying it to our immunological example. The resulting p-values were very similar to those obtained from the generalized Wilcoxon test. We did not conduct our simulation study with skewed data, different error distributions or great outliers due to two reasons. We aimed to include the classical Tobit regression as gold standard which requires strong parametric assumptions as (log) normality and homoscedasticity of the data [10], [11]. Additionally, rank regression is a distribution-free analysis [20]–[22]. Therefore, there was no need to assess the performance of regression models on rank-transformed data when parametric assumptions do not hold.

We confirmed the results of the simulation study by a statistical experiment making use of permutation and bootstraps. We aimed to address accuracy when the Tobit regression on ranks is applied to censored and heavily skewed data that have similar characteristics as cytokine data. The accuracy of the Tobit regression on ranks was of the same magnitude as the accuracy of Tobit regression when the assumptions of normality hold. This confirms that rank regression is a distribution-free statistical method and its concept also works in the setting of heavily skewed censored data.

Compared to the classical Tobit regression there is one limitation: the obtained estimate from the Tobit regression on ranks is the estimate of the rank transformed data instead of the estimate of the observed values. These values are not directly interpretable. However, analogue to log-transformed data rank transformed data may be retransformed to the original observed values if required [22]. After retransformation the obtained estimate represents the median of the data while in classical regression the obtained estimate represents the mean of the data.

### Conclusion

It is highly important to treat gene expression data (expressed as Δct values) as censored when the cycle threshold of a real-time RT-PCR measurement exceeds a certain value and thus, is not quantifiable any more. Applying appropriate statistical methods is crucial. While immunological data at one detection limit (LUMINEX) can be analyzed by standard methods we recommend the Kaplan-Meier method and the generalized Wilcoxon test in order to produce summary statistics and comparison of simple group differences for real-time RT-PCR data expressed as Δct. For multivariate comparison that allows adjusting for potential confounding and interaction effects in large and complex immunological datasets we suggest to apply the Tobit regression on ranks which assumes no underlying distribution of the data and is available in standard statistical packages like SAS. All methods described here may be transferred to other fields of research whose data are affected by similar characteristics such as non-normal distribution and censoring.

## Supporting Information

Figure S1 **Typical distribution of left censored cytokine (real data).**
(TIF)

Figure S2 **Variable after quadratic transformation from normality into a heavily skewed variable.** The distribution has two local maxima and resembles distribution from Fig. S1.
(TIF)

Supplement S1 (DOCX)

Table S1 **Survival function according to the Kaplan-Meier method applied to example with right censoring at multiple detection levels for unexposed children.**
(DOCX)
