# Supplementary figures and images for: Novel Statistical Approaches for Non-Normal Censored Immunological Data: Analysis of Cytokine and Gene Expression Data
Source: PLoS One. 2012 Oct 26;7(10):e46423. doi: 10.1371/journal.pone.0046423 (PMC3482200; doi:10.1371/journal.pone.0046423)

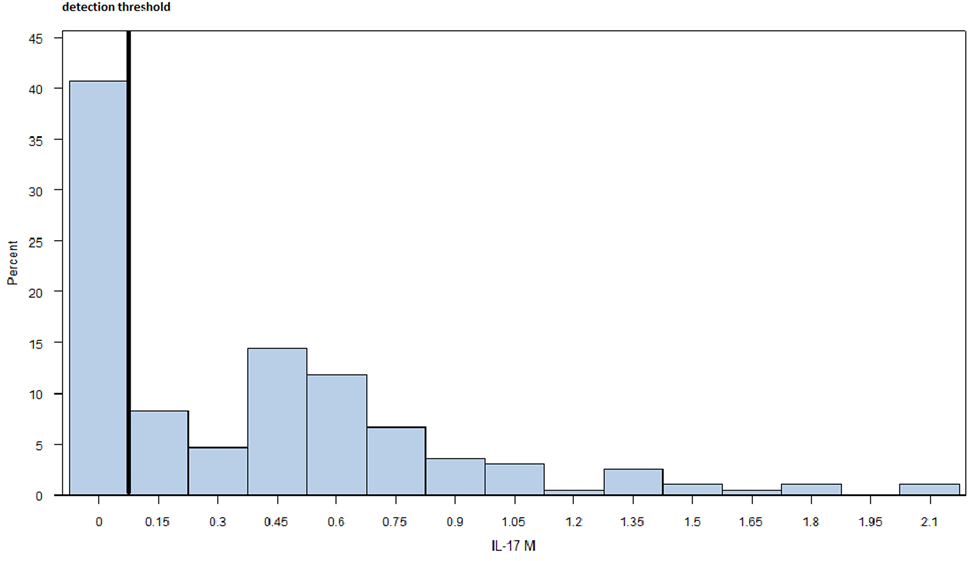

Supplement: Figure S1 — Typical distribution of left censored cytokine (real data). (TIF) [file pone.0046423.s001.tif]

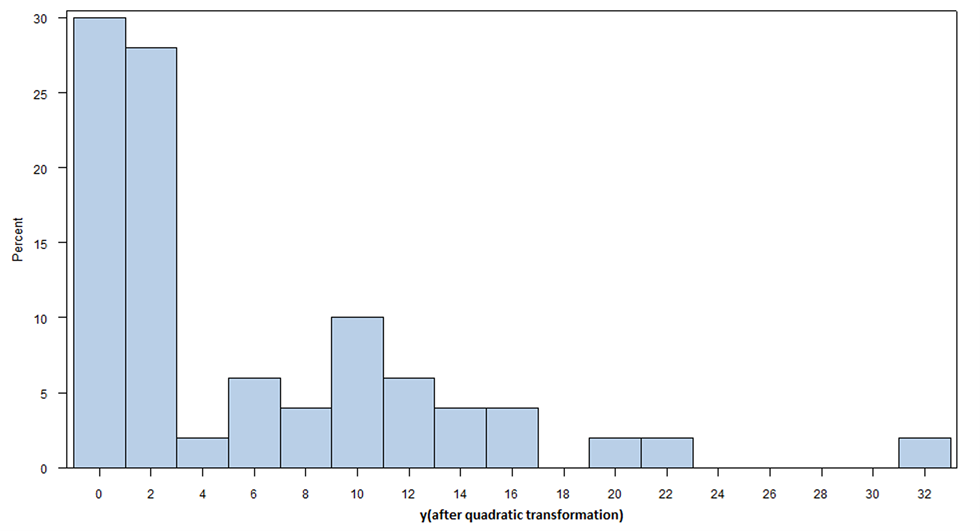

Supplement: Figure S2 — Variable after quadratic transformation from normality into a heavily skewed variable. The distribution has two local maxima and resembles distribution from Fig. S1. (TIF) [file pone.0046423.s002.tif]
