# Supplementary material for: Novel Statistical Approaches for Non-Normal Censored Immunological Data: Analysis of Cytokine and Gene Expression Data
Source: PLoS One. 2012 Oct 26;7(10):e46423. doi: 10.1371/journal.pone.0046423 (PMC3482200; doi:10.1371/journal.pone.0046423)
Supplement: Supplement S1 — (DOCX) [file pone.0046423.s003.docx]

**Supplement S1**

**Novel statistical approaches for non-normal censored immunological data: analysis of cytokine and gene expression data**

***Nikolaus Ballenberger, Anna Lluis, Erika von Mutius, Sabina Illi, Bianca Schaub***

**Supplement Methods:**

**Kaplan-Meier method**

Before the median and the corresponding confidence interval can be derived a life table and a survival plot of the survivor function has to be created. Table S1 shows how the survivor function according to the Kaplan-Meier method was applied to the Δct data in the unexposed group. The first column represents the measured cytokine concentration of the observation expressed as Δct. The next column shows the number of censored measurements with given Δct value. In the third column the number of detected (uncensored) measurements with given Δct value is presented. The column (Number Left) depicts the number of observations left that exceed the given Δct value. The incremental probability is the probability of the observations left (NL) to exceed the cytokine concentration of the given observation. In case of censoring the incremental probability is not calculated, but NL is reduced by NC. The column “survival” presents the product of the incremental probabilities up to that point.

**The Tobit regression on ranks**

The Tobit regression after the economist James Tobin [1] uses three types of information: 1) the observed values above detection limits b) the proportion of data below each detection limit and c) the assumed underlying distribution which may be e.g. normal or log-normal. The Tobit regression estimates a regression model for the data above the detection level, and assumes the same distribution of errors for the censored data (below DL) as for the observed data. The Tobit regression is applicable for both left and right censoring and both single or multiple detection levels.

The Tobit regression can be described in terms of a latent variable$y*$. Suppose

$y*=\alpha+\beta x+\varepsilon$ where $\varepsilon\sim N(0, \delta^{2}\text{) }$

and the observed variable y satisfies:

$$y = y* if \text{ }y> a$$

$y=a if y* <=a$ where a=detection limit.

The disadvantage of this method is that it is vulnerable to violation of the statistical assumptions like normality of the error distribution. E.g. in the presence of heteroscedasticity the estimates of Tobit regression may become inconsistent [2, 3].

Tobit regression on ranks is not calculated on the real values of the observations but on their corresponding ranks. Conover and Iman showed that calculating parametric tests on rank transformed data produce equivalent results as the corresponding non-parametric test [4, 5]. Thus, according to Iman and Conover the corresponding Tobit regression on ranks is given by:

$$R(y*) = \frac{n+1}{2} + \beta(R(x) -\frac{n+1}{2})$$

$$R(y) = R(y*) if R(y*) > R (a)$$

$$R(y) = R (a) if R(y*) <= R (a)$$

where $R$ is the rank of the corresponding variable.

The ranking is performed according to the following method: the entire set of observations of both dependent and independent variables is ranked from smallest to largest. The smallest observation obtains the rank 1 and the highest observation the highest possible rank. Average ranks are assigned in case of ties. Dichotomous variables are not transformed.

Censoring is taken into account by marking the rank transformed observation as censored.

Ranking in SAS (SAS) is supported by the *proc rank* procedure. The Tobit regression is supported in the *Lifereg* and the *Qlim* procedure in SAS [6].

The calculated estimates of the models that are estimates of the ranks may be retransformed to the actual values according to Iman [7]. As Tobit regression is applicable to both left and right censored data at both single and multiple detection levels the Tobit regression on ranks can be used for LUMINEX analysis as well as for Δct data.

**Simulation study**

In order to check the performance (Type-I error rate and Power) of the Tobit regression on ranks we conducted a simulation study. We simulated 10000 data sets by drawing samples from a hypothetical population similar to the Paulchen with respect to sample size and proportion of censored observations.

We conducted a two group comparison with an interval scaled dependent variable. We assumed normal error distribution as we wanted to include the Tobit regression as the gold standard in our simulation study. Two scenarios were considered: In the first scenario both groups have the same mean of 20 and a standard deviation of 2. Scenario 1 is meant to assess the Type-I error rate. In the second scenario there is a mean difference of 1 (mean= 20 and 21 resp.) and a standard deviation of 2. Scenario 2 is meant to assess the power. In each scenario we considered three different sample sizes per group (25, 50 and 100 resp.). Additionally, we added decreasing detection levels to each scenario starting from y=DL if y>26. The next run had y=DL if y>25 until y=DL if y>18. That way the proportion of censoring was always higher in the group with the higher mean.

In the simulation study we compared four statistical models and the standard Wilcoxon rank-sum. Both the models and the standard Wilcoxon rank sum test are considered to be appropriate according to the literature [8]:

1. Quantile regression [9]: In contrast to the [method of least squares](http://en.wikipedia.org/wiki/Method_of_least_squares) as used in linear regression that approximates the conditional *mean* of the response variable given certain values of the predictor variables, quantile regression approximates either the [median](http://en.wikipedia.org/wiki/Median) or other [quantiles](http://en.wikipedia.org/wiki/Quantiles) of the response variable.
2. Logistic regression: logistic regression is a model from the family of the generalized linear models. It is used for prediction of the probability of an event occurring by fitting data to a logit function. From the probability of occurring an event the odds ratio can be computed. Logistic regression describes the relation between one or more independent variables (e.g., age, sex, etc.) and a binary response variable (e.g. yes/no). The cut-off value for dichotomizing the data in our simulation study was the median. When the proportion of censoring was ≥ 50 % we used the detection level as the cut-off value.
3. The Wilcoxon rank-sum test: This test is a [non-parametric](http://en.wikipedia.org/wiki/Non-parametric_statistics) [statistical hypothesis test](http://en.wikipedia.org/wiki/Statistical_hypothesis_testing) and thus assumes no certain distribution of the data. It can be applied here as the detection occurs at a single level.
4. The Tobit regression: the Tobit regression as described above was used in our simulation study as the gold standard.
5. The Tobit regression on ranks as described above.

**Balanced accuracy achieved by permutation and bootstrapping**

We performed this experiment in order to address accuracy in a simple two group comparison. Based on left censored heavily skewed cytokine data (real data in Fig. S1), we created a variable with similar properties: We transformed a normally distributed variable (mean (group 1) = -1.5, mean (group 2) = 0, standard deviation (both groups) = 2) into a heavily skewed variable with two local maxima by quadratic transformation (Fig.S2).

Additionally, we added increasing proportions of left censoring data. First, we assessed the degree of accuracy of the Tobit model on ranks when performed on a heavily skewed variable in comparison to the Tobit model when applied to a normally distributed variable. Second, we investigated the accuracy of the Tobit model when applied to the heavily skewed variable. As a measure of accuracy we chose the balanced accuracy [10].

Specificity was achieved by permutating the grouping variable. To each permutated sample the respective statistical models were applied. The proportion of significant test results out of 1000 tests was calculated. Consequently, all significant findings are the result of random sampling. Sensitivity was assessed by bootstrapping. To each bootstrapped sample the statistical models were applied. The proportion of significant test results out of 1000 tests was calculated. Left censoring was stepwise added until a proportion of 60% of left censoring was reached. At each censoring level the accuracy was calculated.

**References**

1. Tobin J (1958) Estimation of Relationships for Limited Dependent Variables. Econometrica 26(1): 24-36.
2. Arabmazar A, Schmidt P (1982) An Investigation of the Robustness of the Tobit Estimator to Non-Normality. Econometrica 50(4): 1055-1063.
3. Austin P, Escobar M, Kopec J (2000) The use of the Tobit model for analyzing measures of health status. Quality of Life Research 9(8): 901-910.
4. Conover WJ (1980) Practical Nonparametric Statistics New York: John Wiley and Sons.
5. Conover WJ, Iman RL (1981) Rank Transformations as a Bridge Between Parametric and Nonparametric Statistics. The American Statistician 35(3): 124-129.
6. SAS Institute (2009) SAS 9.2 for windows. Cary, NC: SAS Institute Inc.
7. Iman RL, Conover WJ (1979) The Use of the Rank Transform in Regression. Technometrics 21(4): 499-509.
8. Helsel DR (2005) Nondetects and Data Analysis. Hoboken: John Wiley & Sons.
9. Koenker R, Hallock KF (2001) Quantile Regression. The Journal of Economic Perspectives 15(4): 143-156.
10. Brodersen KH, Cheng Soon O (2010). The Balanced Accuracy and Its Posterior Distribution. Pattern Recognition (ICPR), 2010 20th International Conference on.
