# Supplementary material for: Novel Statistical Approaches for Non-Normal Censored Immunological Data: Analysis of Cytokine and Gene Expression Data
Source: PLoS One. 2012 Oct 26;7(10):e46423. doi: 10.1371/journal.pone.0046423 (PMC3482200; doi:10.1371/journal.pone.0046423)
Supplement: Table S1 — Survival function according to the Kaplan-Meier method applied to example with right censoring at multiple detection levels for unexposed children. (DOCX) [file pone.0046423.s004.docx]

**Table S1: Survival function according to the Kaplan-Meier method applied to example with right censoring at multiple detection levels for unexposed children**.

| **Δct -value** | **Number Censored (NC)** | **Number Detected (ND)** | **Number Left (NL) = NL(before) – (NC(before)+ND(before))** | **Incremental probability**  **P=(NL-ND)/NL** | **Probability of exceeding Δct value S=P*S(before)** |
| --- | --- | --- | --- | --- | --- |
| 0 | 0 | 0 | 40 | (40-0)/40=1 | 1 |
| 14.45 | 1 | 0 | 40 | - | - |
| 15.70 | 0 | 1 | 39 | (39-1)/39=0.97 | 0.974*1.00=0.97 |
| 16.80 | 0 | 1 | 38 | (38-1)/38=0.97 | 0.97*0.97=0.95 |
| … | | | | | |
| 20.15 | 0 | 1 | 15 | (15-1)/15=0.93 | 0.933*0.516=0.48 |
| … | | | | | |
| 23.30 | 1 | 0 | 3 | - | - |
| 23.70 | 0 | 1 | 2 | (2-1)/2=0.50 | 0.50*0.177=0.08 |
| 25.05 | 1 | 0 | 1 | - | - |

**Δct** value: measured cytokine concentration of the observation.

Number censored: number of censored measurements with given Δct value

Number detected: number of detected (uncensored) measurements with given Δct value

Number Left: number of observations that exceed given Δct value

Incremental probability: probability of the NL to exceed the Δct of the given observation. In case of censoring the incremental probability is not calculated, but NL is reduced by NC

Probability of exceeding Δct value: the product of incremental probabilities up to that point
